# Immunogenicity of Biotherapeutics: Causes and Association with Posttranslational Modifications

**DOI:** 10.1155/2016/1298473

**Published:** 2016-06-29

**Authors:** Anshu Kuriakose, Narendra Chirmule, Pradip Nair

**Affiliations:** Biocon Research Limited, Research & Development, Bangalore, Karnataka 560099, India

## Abstract

Today, potential immunogenicity can be better evaluated during the drug development process, and we have rational approaches to manage the clinical consequences of immunogenicity. The focus of the scientific community should be on developing sensitive diagnostics that can predict immunogenicity-mediated adverse events in the small fraction of subjects that develop clinically relevant anti-drug antibodies. Here, we discuss the causes of immunogenicity which could be product-related (inherent property of the product or might be picked up during the manufacturing process), patient-related (genetic profile or eating habits), or linked to the route of administration. We describe various posttranslational modifications (PTMs) and how they may influence immunogenicity. Over the last three decades, we have significantly improved our understanding about the types of PTMs of biotherapeutic proteins and their association with immunogenicity. It is also now clear that all PTMs do not lead to clinical immunogenicity. We also discuss the mechanisms of immunogenicity (which include T cell-dependent and T cell-independent responses) and immunological tolerance. We further elaborate on the management of immunogenicity in preclinical and clinical setting and the unique challenges raised by biosimilars, which may have different immunogenic potential from their parent biotherapeutics.

## 1. Introduction

Posttranslational modifications (PTMs) refer to enzymatic modifications that occur after translation, and which result in mature protein products. PTMs increase the functional diversity of the proteome, by the covalent addition of functional groups, proteolytic cleavage of regulatory subunits, or selective degradation of entire proteins. These modifications include glycosylation, acetylation, acylation, ADP-ribosylation, amidation, *γ*-carboxylation, *β*-hydroxylation, disulfide bond formation, phosphorylation, proteolytic processing, and sulfation and influence almost all aspects of normal cell biology and pathogenesis. Therefore, all living cells are tuned to use PTMs to regulate cellular activity. In addition, these PTMs have a major impact on evolution; multisite PTMs lead to a combinatorial explosion in the number of potential molecular states. Such complexity may provide the foundation for sophisticated forms of cellular information processing that are essential for the emergence of complex organisms [1].

In comparison with small molecule drugs, protein pharmaceuticals are complex entities; and since they are usually expressed in cellular systems, they are exposed to factors which could influence PTMs. The PTM profile is dependent on several factors including the type and differentiation status of the host cell, upstream and downstream manufacturing process, formulation, and storage conditions and microheterogeneities formed during fermentation and downstream processing. Naturally occurring PTMs have been associated with unwanted immunogenicity and autoimmune diseases. Recent studies have identified anticitrullinated protein antibodies, along with other antibodies to specific posttranslational modified proteins, as biomarkers in rheumatoid arthritis, psoriatic arthritis, periodontitis, and osteoarthritis [2]. It is hypothesized that such PTMs induce neoepitopes that can generate novel antibody specificities probably triggering autoimmunity. Given the multiplicity of possible PTMs, any variation in a recombinant protein's PTM profile relative to the natural product might be of concern and should be evaluated. Adverse immune reactions can lead to clinical consequences, such as anaphylaxis, reduced drug half-life, and neutralization of the therapeutic protein as well as its endogenous human homologue [3, 4]. However, it should be noted that, of the very large number of patients treated with biotherapeutic proteins over the years, only a few are affected by undesirable immune responses [5, 6]. For example, a ~2% incidence of adverse reactions (attributable to anti-drug antibodies, ADAs) for insulin and an even rarer but more clinically serious effect for erythropoietin has been reported [7, 8]. These studies clearly indicate that some patients are more susceptible to immune responses than others.

In this review, we will identify some of the causes of immunogenicity in therapeutic proteins and will discuss the association of immunogenicity with PTMs, with other critical quality attributes of protein therapeutics, and with patient characteristics.

## 2. Immunogenicity and Its Causes

Immunogenic response to therapeutic molecules can generate anti-drug antibodies (ADAs), which can be either neutralizing or nonneutralizing. Neutralizing antibodies (NAbs) bind to sites in therapeutic proteins in such a way that they directly impair or abrogate the biological functions of therapeutic proteins [6, 9]. NAb responses have the potential to cause negative clinical consequences by neutralizing the therapeutic product and therefore reducing efficacy, as has been seen with factor VIII or streptokinase [10, 11]. This reduced efficacy would, in some cases, require the need to dose patients more frequently to get the desired clinical effect. The situation can further be aggravated if the NAbs neutralize not only therapeutic proteins but also the endogenous counterpart of the therapeutic agent, resulting in severe adverse consequences. Examples of drugs inducing ADAs which also inactivate autologous proteins include recombinant human thrombopoietin [12, 13], erythropoietin [5, 14, 15], GM-CSF [16], and many interferons [17–19]. The problem is most often seen with non-mAb therapeutic proteins with significant similarity to host proteins (except for a few amino acid changes or glycosylation differences) [13]. However, some non-mAb proteins such as insulin, factor XIII, and *α* interferons (IFNs) primarily induce nonneutralizing ADAs and the effect is not physiologically debilitating. In these cases, clinicians often continue treatment in the presence of ADAs. This may indicate that it is not always possible to break self-tolerance even when the self-protein is exogenously chronically administered. Overall, only a small percentage of treated patients develop adverse immunogenic reactions attributed to the formation of NAbs [6].

Most patients who develop ADA response to therapeutic proteins generate nonneutralizing antibodies (NNAbs). These antibodies bind to antigenic sites in the therapeutic proteins in ways that do not affect the therapeutic effects of these drugs. Examples are NNAbs generated against tumor necrosis factor receptor and recombinant human growth hormone [20]. In some cases NNAbs can accelerate the clearance of therapeutic proteins resulting in reduced drug efficacy [13]. Product- and process-related factors can affect immunogenicity by minor alterations in the tertiary structure of the molecule such as altered protein folding. Additionally, patient characteristics, dose, and route of administration of the biotherapeutics can also lead to an increased risk of immunogenicity [21]. This will be described in more detail in later sections.

### 2.1. Product- and Process-Related Causes of Immunogenicity 

The first therapeutic insulin products in the 1920s were of bovine or porcine origin and were therefore immunogenic in humans. In some cases, fatal anaphylactic reactions were reported [22]. The molecular structure of proteins purified from animal sources is different from that of their human counterparts. Thus it is expected that these proteins will be seen as “foreign” by the human immune system. Interestingly, removal of proinsulin, C-peptide, glucagon, and somatostatin from porcine insulin preparations led to a remarkable decrease in immunogenicity [22]. These results suggested that the anti-insulin antibodies generated may have been against noninsulin proteins or adjuvant-like contaminants [23]. This observation indicated that deviation from the structure of the human homologue is not the only determinant of immunogenicity.

Impurities have been held responsible for the immunogenicity of several therapeutic proteins. Human growth hormone (hGH) derived from the pituitary glands of cadavers and from patients undergoing hypophysectomy had been used in hypopituitary children to stimulate their growth [43]. Fifty percent of treated children developed immune reaction to the first clinical grade hGH; and this was attributed to the presence of 40% to 70% aggregated hGH in the product [44]. Improvement of the purification process decreased the aggregates to less than 5%, which resulted in slower onset of antibody production. Resulting antibodies had high affinities but were significantly less persistent [4].

Humanization of monoclonal antibodies has significantly decreased immunogenicity, especially the intense immunogenicity (allergic reactions culminating in anaphylactic shock) observed with early murine antibodies, which generated a human anti-mouse antibody (HAMA) response. However, some humanized and even fully human sequence-derived antibody molecules still carry immunological risk. Often the cause for immunogenicity with these fully human molecules is associated with unique (nonhuman) sequences in the cluster of differentiation regions (CDRs) of these antibodies [24] and modifying certain amino acids in these regions could reduce immunogenicity risk. For example, despite being humanized, alemtuzumab induced binding antibodies and NAbs in 30% to 70% of patients. It was shown that pretreatment with an altered version of alemtuzumab, which no longer binds to its target, induced immunogenic tolerance to alemtuzumab itself [28]. Other fully human antibodies such as canakinumab, ofatumumab, and pembrolizumab induced ADAs at very low incidence (<0.5%) [30, 35, 39, 45]. Details of immunogenicity and NAbs reported for mAbs are shown in Table 1.

Intrinsic factors influence the immunogenicity of antibodies; for example, antibodies directed at cell surface markers are deemed to have a higher risk of immunogenicity than those against soluble factors [24]. The reasons for this are not completely understood but may include antigen internalization and subsequent processing and presentation by target cells [24, 46]. A similar intrinsic factor is the presence of carbohydrate side-chains attached to the antibody via glycosylation sites, conferred by the amino acid sequence of the light chain constant region, the heavy chain constant region, or the V region itself [47, 48]. The presence of a galactose-alpha-1,3-galactose sugar within a carbohydrate structure on the Fab fragment of cetuximab was found to be associated with severe anaphylactic reactions to the antibody. Most patients were found to have preexisting immunoglobulin E (IgE) antibodies specific for the galactose-alpha-1,3-galactose sugars [49]. Notably, when cetuximab was manufactured in a cell line that could not add galactose-alpha-1,3-galactose to the antibody carbohydrate (a Chinese hamster ovary- [CHO-] derived manufacturing cell line), the product was much less immunogenic [49].

Other PTMs, such as glycation, deamidation, and oxidation of amino acid side-chains, may confer immunogenicity as well [24, 50]. Introducing additional N-linked glycosylation sites to create an erythropoietin (EPO) product with improved efficacy and catabolic half-life [51] did not result in increased immunogenicity [52]. However, it was noted that the subcutaneous administration of EPO was associated with pure red cell aplasia (PRCA) in some cases, because of NAbs generated to endogenous EPO. Although the cause of this reaction is still uncertain [53], extractables and leachates from the container are thought to have been responsible [54]. An increased concentration of anti-ESA IgG4 antibody is associated with the development of antibody mediated PRCA (amPRCA) [55].

### 2.2. Patient-Related Causes of Immunogenicity and Influence of Route of Administration 

Generally, patients with impaired immune systems (e.g., cancer patients receiving chemotherapy) may be less likely to develop antibodies to therapeutic proteins than immunocompetent individuals [56, 57].

Exposure of patients to replacement therapeutic proteins that the patients cannot synthesize frequently leads to the generation of NAbs [58]. For example, NAbs frequently develop in severe congenital factor (F) XI-, FIX-, and FVIII-deficient patients treated with the missing therapeutic proteins [10, 59–61]. The inherent polymorphism of human proteins may be another contributing factor toward immunogenicity. This is especially applicable in hemophilia, where patients are predominantly categorized into two major groups. One group has large deletions, nonsense mutations, and intron 22 inversions leading to nondetection of FVIII protein in the plasma. This group, who have never been exposed to FVIII, generate anti-FVIII in more than 30% of treated patients. The second group of patients have small deletions, insertions, and point mutations and therefore might be innately tolerant to therapeutic FVIII administration. In this latter group, anti-FVIII antibody prevalence is less than 10% [62]. When mutation types are further subdivided based on their risk of antibody formation, patients with large deletions have highest risk (~75%), followed by patients with nonsense mutations and those with inversions of intron 22 [63–65]. Since small deletion/insertion mutations cause a frameshift, resulting in subsequent stop codon and truncated protein, a risk similar to that of a major deletion might be expected. Surprisingly, these patients had a relatively low risk (7.5%) [60]. This was attributed to polymerase errors which lead to restoration of reading frames for small deletion/insertion mutations. The small amount of endogenous FVIII produced was apparently sufficient to generate tolerance [63, 66]. However, mutations in the FVIII gene do not completely explain immunogenicity to therapeutic FVIII. Only one-third of the patients with intron 22 inversion develop anti-FVIII antibodies. Speculations explaining this include presentation of maternal FVIII to the fetal immune system to induce immunogenic tolerance and polymorphism in the immune systems of patients, which either hinders or synergizes anti-FVIII antibody formation [64, 67].

A meta-analysis conducted by Scharrer et al. indicated influence of race on the risk of antibody formation [68]. The incidence of antibody formation in African-Americans was double that in Caucasians (51.9%, 14 of 27, versus 25.8%, 51 of 191). This again indicates that genetic polymorphism has a role in immunogenicity. The incidence of antibody formation in siblings (50%) is significantly higher than in extended hemophilia A relatives (9%), suggesting that sibling risk is correlated [69]. However, nongenetic factors could also influence immune response as variations in response in monozygotic twins have been described [70]. Since the genetic defects in FVIII are assumed to be similar in siblings and extended relatives, polymorphisms in immune response genes may influence the risk of anti-FVIII antibody formation. An analysis of MHC class I and II alleles identified A3, B7, C7, DQA0102, DQB0602, and DR15 as risk alleles (relative risk: 1.9–4.0). MHC class I/II alleles C2, DQA0103, DQB0603, and DR13 were identified as protective alleles (relative risk 0.1–0.2) since they occurred more often in patients who did not develop antibodies than in those who did. However, sample sizes were generally too small for statistical confirmation of differences.

Immunogenicity is associated with the route of administration of a therapeutic molecule. The skin and the mucosal membranes make up the primary surface barriers to pathogens, and just beneath these lies the primary machinery to protect the body when these barriers are breached, abundant professional antigen-presenting cells (APCs). Routes of administration that involve the skin or mucosa thus may carry the greatest potential for an immunological response. Interestingly, exposure via gut mucosa is generally tolerogenic; however the challenge is the effective oral delivery with bioavailability of the biotherapeutic [71]. The probability of immune response is highest after subcutaneous injection, followed by intramuscular, intranasal, and intravenous routes. Subcutaneous administration generally localizes and prolongs the exposure of the protein to a small area within close proximity of lymph nodes, where B and T cells are present [72]. Lymphatic uptake can enhance exposure to APCs. Dendritic cells may potentially be activated if an adjuvant-like factor (e.g., impurities, host cell proteins, or endotoxin) or a danger signal is present. Peng et al. showed that changing the route administration reduces the frequency of ADAs [73, 74]. Extensive clinical experience with pulmonary delivery of biotherapeutic insulin showed that patients with type 1 and type 2 diabetes switching from subcutaneous dosing resulted in larger ADA responses [75, 76]. The antibodies were of the immunoglobulin G (IgG) class, were not neutralizing, and had no impact on clinical efficacy and safety. The importance of route of administration is not accepted by all; Schellekens argues that the immune reaction is not predicated by route of administration but rather is inherent in a therapeutic molecule itself [77].

Generally, short-term therapy is less likely to be immunogenic than long-term therapy, although intermittent treatment is more likely to elicit a response than continuous therapy [78, 79]. Also, lower doses are generally more immunogenic than higher doses, as typically seen with mAbs where the phenotype is more tolerogenic. This may be because of the evolution of the immune system to be generally less tolerant of low-abundance proteins. This was observed in a primate study of adalimumab where 16/16 monkeys developed PAHA response at low dose while only 2/16 animals developed PAHA response at high dose [72]. Therefore in clinical practice, high-dose regimens are used as a mode of therapy to induce tolerance (e.g., for factor VIII) [80].

## 3. Mechanisms of Immunogenicity of Biotherapeutics

The immune system can generate antibodies to therapeutic proteins by two general mechanisms: one relies on T cell costimulation of B cells while the other is independent of T cell [58].

### 3.1. T Cell-Dependent Immune Response 

Analysis of antibodies from clinical studies suggests that serious side effects are mainly driven by high levels of IgG antibodies, suggesting a T cell-dependent pathway. In fact, IgG antibodies make up the majority of the ADA responses [81]. Naïve B cells require two signals for their proliferation and differentiation into antibody secreting plasma cells. The first signal is generated by the direct binding of the antigenic protein to B cell receptors on naïve B cell surfaces. This protein is then internalized, processed, and returned to the surface as peptides bound to the MHC class II molecules. The second signal is delivered by the armed T helper (Th) cells, which recognize the same antigen (or a peptide within the antigen, a concept known as linked recognition), via the binding of the T cell receptors (TCRs) to the peptide: MHC class II complex on the surface of naïve B cells. Another interaction is governed by the binding of B7 on B cells to CD28 on T cells. B-T cell contact leads to the overexpression of the B cell costimulatory molecule CD154 (CD40L) on the Th cell surface and secretion of B cell stimulatory cytokines (IL-4, IL-5, and IL-6) by the Th cells. This in turn activates the B cells and leads to their differentiation into antibody-secreting (short- and long-lived) plasma B cells. Some of these activated B cells also become memory cells, which maintain the pool of long-lived plasma cells and react rapidly to rechallenge by producing short-lived plasma cells. This T cell dependent immune response is thus usually long-lasting and of high titer, particularly for foreign or exogenous proteins [81].

### 3.2. T Cell-Independent Immune Response 

In the T cell-independent antibody response, the ability to bypass Th cell costimulation leads to a more rapid antibody response. This type of response is typically evoked by particulate antigens and sequences of microbial and viral origin [6] (repetitive epitopes termed* pathogen associated molecular patterns*). Antigens that are expressed on the surface of pathogens in an organized, highly repetitive form can activate specific B cells by cross-linking of antigen receptors in a multivalent fashion [82]. This activation is dependent on the formation of a small number of antigen receptor clusters, each of which contains approximately 10 to 20 antigen-bound membrane Ig (mIg) molecules [82]. These clusters induce local membrane association of multiple activated Btk (Bruton's tyrosine kinase) molecules, which results in long-term mobilization of intracellular ionized calcium. Such persistent calcium fluxes efficiently recruit transcription factors, and thereby induce T-cell-independent B cell activation and proliferation. While this first signal of multivalent mIg cross-linking can induce B cell proliferation, a second signal in the form of engagement of members of the Toll-like receptor (TLR) family could selectively induce Ig secretion in B cells that were activated by multivalent, but not by bivalent, antigen receptor engagement. Due to the lack of affinity maturation, this pathway typically results in an IgM-type response, which is transient, of low titer, and of poor specificity [72]. Changes to the structure of a therapeutic protein may alter its miscibility in ways that enhance aggregation or cause it to resemble a pathogen, thereby greatly increasing antigenicity.

Typically, an immune reaction can be triggered by most therapeutic proteins inducing antibody responses. Based on the trigger, the immune reaction can vary from low-titer, low-affinity, transient IgM antibody responses to high-titer, high-affinity responses, followed by class switching and IgG responses. Consequences of this transition can range from minimal to severe and life-threatening [72].

## 4. Posttranslational Modifications and Their Correlation with Immunogenicity 

Most therapeutic proteins are synthesized in the endoplasmic reticulum (ER) and are eventually secreted. While some modifications occur before the proteins are secreted, others happen afterwards, during* in vitro* processing, including purification, formulation, and storage, and during administration into patients [83].

Modifications of proteins that occur in the ER, golgi, and exocellular spaces have been reviewed in detail by Fineberg et al. [75]. These modifications are disulphide bond formation, gamma carboxylation of glutamate residues, and beta hydroxylation of aspartate and asparagine residues in the ER; tyrosine sulfation, propeptide processing, O-linked glycosylation, phosphorylation, and amidation in the golgi; and deamidation, glycation, N-terminal pyroglutamate formation, oxidation, and proteolytic processing in the exocellular spaces. Here, we will discuss protein structure, glycosylation, and chemical modifications.

Posttranslational modifications can have direct or indirect effects on immunogenicity. The modified part of the biotherapeutic itself could induce an immune response, or its presence can affect the tertiary structure of the protein subtly causing the biotherapeutic to become immunogenic [4].

### 4.1. Protein Structure 

Primary amino acid sequence can affect protein structure, and hence immunogenicity, as is observed with animal-derived insulins [22]. For similar reasons, immunogenicity was higher for the first murine therapeutic antibodies, as compared to later chimeric, humanized, or fully human antibodies [84]. It is very interesting to note that while there are only 20 standard amino acids (19 amino acids and 1 imino acid), there are about 200 different functional amino acids after hydrolysis. The role of PTMs is thus significant [83]. Over the years, a significant number of modifications have been identified and several of them characterized [3, 85]. New epitopes in protein structure may be created due to the chemical modification of the protein, whereby new covalent crosslinks between amino acid residues are formed. These new protein structures could lead to the formation of aggregates, which may contain danger signals that greatly enhance immunogenicity.

### 4.2. Glycosylation 

Glycosylation is the covalent addition of carbohydrate molecules (glycans) to the protein surface. It is the most common, complex, and heterogeneous PTM that can occur in both endogenous and therapeutic proteins [3, 86]. Almost half of the therapeutic proteins that are approved or in clinical trials are glycosylated [87]. The considerable heterogeneity in glycosylation profile of products can arise from the differences in the glycan itself (type, structure) or from the attachment pattern (site, extent of occupancy of possible sites). These variabilities may depend on the production and purification process [88]. Since glycans can influence the physicochemical (e.g., solubility, electrical charge, mass, size, folding, and stability) as well as the biological (e.g., activity, half-life, and cell surface receptor function) properties of proteins [89], any change with respect to the production or purification process, even in cell line, can alter glycosylation, thereby potentially altering physiological effects [4]. Glycosylation can have a direct or indirect impact on the immunogenicity of therapeutic proteins as well. The glycan structure itself can induce an immune response, or its presence can affect protein structure in such a way that the protein becomes immunogenic. Recent advances in analytical abilities, including matrix-assisted laser desorption ionization (MALDI), electrospray ionisation mass spectrometry (ESI-MS), and novel fluorescent tags for high performance liquid chromatography (HPLC), can help in effectively characterizing and picking up potential changes in glycan profile of therapeutics [90].

Over the past decade, at least four nonhuman carbohydrate structures that are able to induce an immune response in humans have been identified. They are galactose-*α*1,3-galactose (*α*-Gal epitope), N-glycolylneuraminic acid (Neu5Gc epitope), *β*1,2-xylose (core-xylose epitope), and *α*1,3-fucose (core-*α*1,3-fucose epitope) [4], of which the first two are well studied and are described here. The first observations of immune reactions against *α*-Gal and Neu5Gc were described in the context of xenotransplantation of pig organs in humans [91] and the targeting of vaccines to APCs in cancer immunotherapy. Autologous tumor cell membranes from solid tumors are processed to express *α*-Gal epitopes by incubation with neuraminidase, recombinant alpha1,3GT, and uridine diphosphate galactose [92].

Recently, the presence of *α*-Gal and/or Neu5Gc was demonstrated in several therapeutic mAbs [4], including cetuximab, a chimeric mouse-human IgG1 monoclonal antibody approved for use in colorectal cancer and squamous-cell carcinoma of the head and neck [4]. About 3% of patients develop severe hypersensitivity reactions within minutes after the first exposure to cetuximab, and a higher prevalence (up to 33%) may be seen in certain geographical regions. Most patients with hypersensitivity possess IgE antibodies against cetuximab before the start of therapy. These antibodies were found to be specific for the *α*-Gal epitope and related to IgE antibodies involved in anaphylactic reactions to red meat [4, 93]. All humans have IgA, IgM, and IgG antibodies against *α*-Gal, representing approximately 1% of circulating immunoglobulin. To our knowledge, the presence of IgA, IgM, and IgG antibodies against *α*-Gal did not correlate with accelerated clearance of cetuximab. Life-threatening hypersensitivity reaction with cetuximab was associated with preexisting IgE anti-*α*-Gal antibodies [94]. Qian et al. [95] demonstrated that the *α*-Gal epitopes are located in the Fab regions of the cetuximab antibody. The intravenous injection method and the presence of *α*-Gal on both Fab regions, which enables efficient cross-linking of IgE on mast cells, may explain the prompt immune reaction to cetuximab in a certain patient subset. The murine cell line SP2/0 used to produce cetuximab expresses the gene encoding for *α*1,3-galactosyltransferase, the enzyme responsible for the synthesis of the *α*-Gal epitope. Prevention of incorporation of the terminal *α*-Gal motif in therapeutic mAbs during production could help combat the problem of immunogenicity to an extent. Measures could include knocking out the gene for *α*1,3-galactosyltransferase in murine cells or using another expression system such as the CHO cells which may not produce the *α*-Gal epitope glycoform [96]. Other biotherapeutics like infliximab also have *α*-Gal epitopes located on Fc linked glycans but these were not found to be recognized by IgE anti-*α*-Gal antibodies. The relative low abundance of *α*-Gal epitopes and their location within the Fc region might be possible reasons for this lack of recognition. So far, IgE anti-*α*-Gal antibodies seem to have a significant importance to patients treated with cetuximab [94].

Humans synthesize the sialic acid N-acetylneuraminic acid (Neu5Ac) but are not able to synthesize Neu5Gc [4]. Consumption of Neu5Gc-rich foods, for example, red meat and milk products, allows for the accumulation of Neu5Gc on the surface of epithelial and endothelial cells [93]. As a result, the human immune system recognizes Neu5Gc as foreign and shows high levels of IgA, IgM, and IgG antibodies against Neu5Gc (0.1%–0.2% of circulating immunoglobulin) [93]. Injecting products that contain Neu5Gc in individuals with preexisting antibodies can cause the formation of immune complexes that potentially activate complement or affect half-life of the drug. Ghaderi et al. [97] showed that the clearance of cetuximab increases significantly in mice when anti-Neu5Gc antibodies are preinjected. Maeda et al. [98] detected the presence of the Neu5Gc epitope in three commercial mAb pharmaceuticals produced in murine cell lines (cetuximab, gemtuzumab, and infliximab), whereas it was absent in other mAbs produced in CHO cell lines (tocilizumab, bevacizumab, and adalimumab). CHO cells are reported to be negative for *α*-Gal and Neu5Gc epitopes. However, these cells are capable of taking up these glycoforms from the cell culture media and metabolically incorporating them into the expressed protein [4]. Therefore, in addition to using these cell lines, media and other components should be void of components such as Neu5Gc [97].

Analysis of biotherapeutic mAbs purified from serum of subjects demonstrates that the PTM profile of the protein changes* in vivo*. Examples include deamidation at Asn-33 and oxidation at Trp-105 in the light chain and heavy chains, respectively, of two therapeutic mAbs [99]. Furthermore, a recent study shows that different levels of mannosylation of mAbs can have significant impact on pharmacokinetic parameters, including clearance and area under the curve (AUC) [100]; however, the increase in mannose did not impact immunogenicity rates [101]. Mannose receptors, expressed at high levels on DCs, mediate the capture, processing, and presenting of antigens (mannose-expressing glycoproteins) for an immune response. This response, depending on several factors, could either be immunogenic or tolerogenic [100, 102].

Glycans may also indirectly impact the immunogenicity of biotherapeutics through changes in the folding, solubility, or stability of the proteins. For example, recombinant human IFN *β* produced in* E. coli* is not glycosylated and is prone to aggregation leading to increased immunogenicity, as compared to the recombinant IFN *β* from CHO cells, where glycosylation reduces immunogenicity [4, 103].

### 4.3. Chemical Composition 

Compared to glycosylation, other PTMs are less well understood [104, 105]. A biopharmaceutical may be chemically modified through accidental degradation in one of the many bioprocessing steps: fermentation, virus inactivation, purification, polishing, formulation, filtration, filling, storage, transport, and administration. Chemical modifications during bioprocessing may include deamidation, oxidation, isomerization, hydrolysis, glycation, and C/N terminal heterogeneity of the protein [106]. The susceptibility of an individual amino acid residue to chemical modification is dependent on neighboring residues; tertiary structure of the protein; and solution conditions such as temperature, pH, and ionic strength. Chemical modification may give rise to a less favorable charge, thus leading to structural changes or even the formation of new covalent crosslinks [107]. Covalent crosslinking could enhance immunogenicity by causing aggregation [108–110]. Multiple studies have indicated a strong correlation between aggregates and immunogenicity [89, 111–113]. Deamidation, isomerization, and oxidation have also been associated with potential immunogenicity [4].

Deamidation of proteins accelerates at high temperature and high pH and can occur during bioprocessing and storage. Deamidation of Asn and Gln contributes to charge heterogeneity of therapeutic proteins, determines the irreversible thermal denaturation of proteins at acidic and neutral pH, regulates the rate of protein breakdown, and could shorten* in vivo* half-life. Deamidation followed by isomerization of asparagine to isoaspartate (isoAsp) has been shown to alter protein structure, thereby potentially making the protein immunogenic [114]. Deamidation can be accompanied by some degree of oxidation, conformational changes, and fragmentation and aggregation, again posing a serious risk of enhanced immunogenicity [4].

Oxidative chemical modification of amino acid residues alters secondary and tertiary protein structures. This favors interaction between protein surfaces and subsequently leads to noncovalent aggregation [115]. Studies using metal-catalyzed oxidation (MCO) have shown that therapeutic proteins can aggregate and can also be immunogenic [4, 115]. Chemical stresses during manufacturing and storage can be caused by exposure to light or elevated temperatures and by the presence of oxygen, metal ions, or peroxide impurities from excipients. Trace amounts of iron, chromium, and nickel were found to leach into the formulation buffer via contact with the stainless steel surfaces typically used during bioprocessing [116]. Tungsten oxide-mediated oxidation caused precipitation of monoclonal antibodies and was pH-dependent [117]. Similarly in EPO, aggregation due to tungsten leachates from the container was associated with immunogenicity [54].

Despite limited information on the association of actual chemical modifications during biopharmaceutical manufacturing and immunogenicity, it is always prudent to be prepared for an untoward possibility. Preventative measures should include careful evaluation of buffers, surface materials, and conditions during manufacturing, transport, and storage. Extensive characterization of molecules using techniques like size exclusion chromatography, supported by orthogonal techniques like analytical ultracentrifugation (identifying aggregation) [118], circular dichroism (CD), and intrinsic fluorescence spectroscopy, can indicate deviations from secondary and tertiary structures. These steps incorporated into the process development will help in mitigating risks of immunogenicity.

## 5. Managing Immunogenicity 

### 5.1. Managing Immunogenicity in a Preclinical Setting 

The 2011 ICH S6 Guideline (preclinical safety evaluation of biotechnology-derived pharmaceuticals) describes the need for detection and characterization of antibodies in repeat-dose studies using animal models. However, relevant species must be used for* in vivo* studies, that is, one in which the target epitope is expressed. Immune responses are species-specific; therefore, induction is not entirely predictive of antibody formation in humans [119, 120]. Animal models are constrained by lack of genetic diversity which is a primary factor for diverse immune response frequently observed in human beings [121]. Rodent models for immunogenicity testing are, therefore, less useful than animals that show a higher degree of homology with humans and more genetic diversity than inbred mouse strains, such as nonhuman primates; however, these are not widely used due to ethical constraints. Conventional nontransgenic animal models can be useful for highly conserved proteins, but a lack of immune tolerance to human proteins limits their use for immunogenicity testing. These animal models can be useful for comparing the immunogenicity of two similar products, that is, the immunogenicity of an originator and biosimilar product; this may not reflect the human situation but may provide a warning against advancement of a biosimilar if the immunogenicity profile observed differs from that of the originator.

Despite the limitations associated with the use of animals to predict immunogenicity, several transgenic animal models have been generated for this purpose. Transgenic mice are often the preferred* in vivo* model to predict immunogenicity as they are tolerant to the administered human protein [122, 123] and can be used to study the immunogenicity of biotherapeutic aggregates. In a study by van Beers et al., the IFNb-1a aggregate percentage and extent of denaturation were shown to influence the ability of aggregates to break tolerance in transgenic mice. In these experiments, immune tolerant mice were immunized with IFNb-1a formulations and antibody responses measured. Only noncovalently bound aggregates that retained some native epitopes were able to break tolerance resulting in a transient immune response; removal of aggregates prevented this breakdown of tolerance [123]. Additionally, mice expressing human MHC molecules can be used to compare antibody and T cell responses to vaccines and protein therapeutics [124]. High ADA titers were observed after injection of a metal catalyzed, oxidized, and aggregated IgG1 sample in nontransgenic and transgenic mice [4]. Therapeutic interferons oxidized and aggregated via the same metal-catalysis method were able to overcome the immune tolerance of transgenic mice that were immune tolerant for the administered human proteins [125, 126]. The transgenic mice also developed antibodies against oxidized and aggregated rhIFN*β*-1a treated with H_2_O_2_ [126], but not against oxidized rhIFN*α*-2b treated with H_2_O_2_ [89], probably due to the absence of aggregation. Use of animal models in immunogenicity testing is discussed more extensively in the review by Brinks et al. [121].

*In vitro* techniques can also be used to assess the immunogenic potential of therapeutic proteins. These could be used to predict the risk of immunogenicity in preclinical setting. The expression of APC-surface molecules differs following activation; for example, the expression of MHC (class I and II), costimulatory molecules, and cytokine receptors is enhanced. Flow cytometry is an* in vitro* technique that can be used to determine differences in cell surface molecule expression, indicative of APC maturation that may initiate T cell responses [127, 128]. T cell proliferation assays are also useful tools to study the activation and proliferation of T cells in the presence of antigen [129]. Additionally, the release of immunomodulatory cytokines can be characterized by enzyme-linked immunosorbent assay. This approach can be used to assess the quality of an induced immune response, as specific cytokines can be markers of Th1 (IL-12 and IFN*γ*) or Th2 immunity (IL-4 and IL-10). T cells that respond to a particular epitope* in vitro* can be labeled with MHC class II oligomers and sorted by flow cytometry; the phenotype of responsive T cells can then be determined using intracellular cytokine staining [124, 130]. Human peripheral blood mononucleated cells, when stimulated with aggregated monoclonal antibody, induce an adaptive T cell response characterized by CD4 T cell proliferation and release of cytokines like interleukin- (IL-) 1*β*, IL-6 and TNF*α*. These cytokines can be used as potential biomarkers for aggregate immunogenicity [129]. It should be noted that these* in vitro* techniques may indicate the probability of an immune response for a biotherapeutic but cannot predict its clinical consequences. Correlative studies with marketed biotherapeutics in these assays may refine these methods further, to enable prediction of relevant immunogenicity [4, 127].

In addition to the assays described above,* in silico* techniques have been developed for the prediction of antigenicity by identification of potential T cell epitopes [131].* In silico* methods have been shown to successfully identify MHC class II-restricted epitopes within biotherapeutics [132]. Knowledge of aggregation-prone regions may also help in the design and selection of biotherapeutic candidates and reduce aggregation concerns [133]. For example, aggregation motifs that lack charge have been found in the light chain regions of mAbs, including Erbitux and Raptiva. This computational approach could, therefore, be useful to screen biotherapeutic candidates early in drug development [128].

Overall, preclinical methods have been focused on identifying potential immunogenicity associated with formation of aggregates often considered the “bête noire” for immunogenicity [134]. However, the challenge remains in identifying potential immunogenicity with low levels of aggregation induced naturally by PTMs (as described previously) especially in contexts of process change, shipping, and clinical use. The preclinical techniques to predict immunogenic potential described here are still exploratory. Developing more robust methods to predict possible immunogenicity attributable to PTMs should be the way forward to reduce clinical risk.

### 5.2. Managing Immunogenicity in the Clinic 

Prior to treatment, patients should be screened for established biomarkers to check for potential immunogenicity. A retrospective analysis of cetuximab evaluated whether the presence of pretreatment IgE antibodies against cetuximab is associated with severe infusion reactions (SIRs) during the initial cetuximab infusion. This analysis used 545 banked serum or plasma samples from cancer patients participating in clinical trials. Patients with a positive test indicating the presence of pretreatment antibodies had a higher risk of experiencing an SIR. Although this test had low positive predictive value, it clearly indicated an association between the presences of preexisting IgE antibodies against cetuximab with SIRs, supporting prior association studies [135].

Infantile Pompe disease resulting from a deficiency of lysosomal acid *α*-glucosidase (GAA) requires enzyme replacement therapy (ERT) with recombinant human GAA (rhGAA); immunogenicity can be managed with a combination of rituximab with methotrexate ± intravenous gamma globulins (IVIG). This is an option for tolerance induction of CRIM negative Pompe to ERT when instituted in the naïve setting or following antibody development [136].

**Figure 1:**
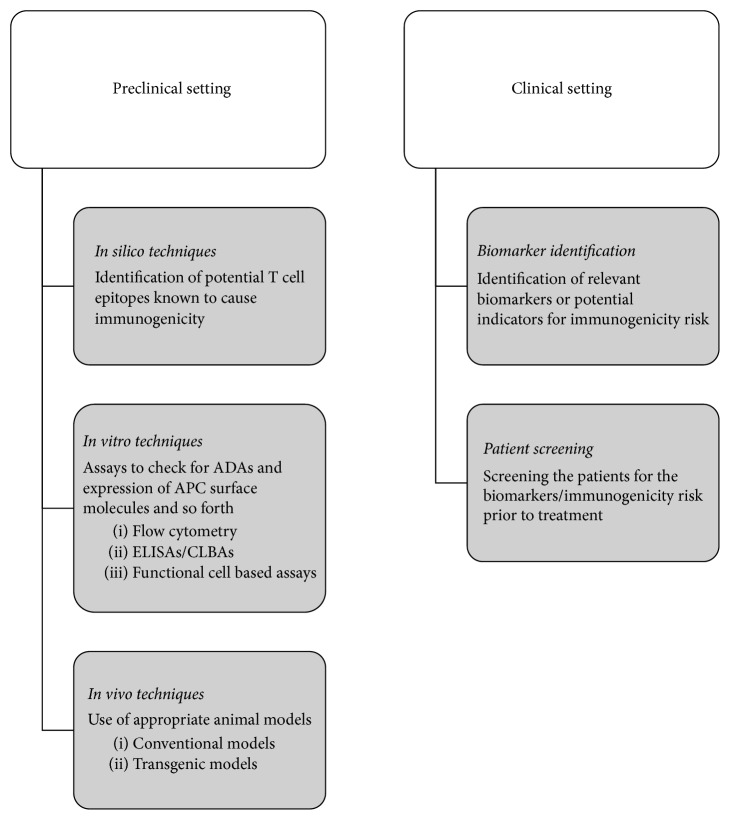
Management of immunogenicity in preclinical and clinical settings. ADAs: anti-drug antibodies; APC: antigen presenting cell; ELISA: enzyme-linked immunosorbent assay; CLBA: competitive ligand binding assay.

With adalimumab, dosing over the NAb response is probably effective in recapturing symptomatic response. In patients with Crohn's disease, adalimumab dose escalation is effective for recapturing symptomatic response after secondary loss of response, but more than half of the patients eventually experience a tertiary loss of response [137]. An additional risk with dosing over the prescribed dose could involve adverse events such as serum sickness and hypersensitivity reactions [138]. Another strategy commonly adopted with anti-TNF therapeutics is to switch the biologic when a patient becomes refractive to a particular anti-TNF. In some cases, suppressing the immune response (formation of ADAs) with mild doses of methotrexate was seen to be beneficial [139].  Figure 1 gives a schematic representation of managing immunogenicity.

### 5.3. Managing Immunogenicity against Biosimilars 

In recent years, follow-on biologics (or biosimilars) and generic protein therapeutics have become more prevalent as the patents associated with the original drugs expire. The first biosimilar reached the market almost a decade ago [140]; and biosimilar use has been steadily rising. Managing immunogenicity arising due to biosimilars is another challenge.

For small molecules approved in the EU, the generic paradigm applies; a product is pharmaceutically equivalent to a competitor molecule when it has the same qualitative and quantitative composition. If the products are shown through pharmacokinetic studies to have the same bioavailability, they are deemed bioequivalent. Generally, this is demonstrated in a limited number of studies in healthy volunteers [141]. Once products are deemed bioequivalent, they are assumed to be therapeutically equivalent and essentially similar in terms of benefits and risks* in vivo*.

However, such paradigm is not applicable for biopharmaceuticals. Biopharmaceuticals are large and intricate molecules and frequently subjected to extensive PTMs that are sensitive to differences in manufacturing conditions [142]. Pharmaceutical equivalence for biopharmaceutical products cannot be directly demonstrated. Therefore, the biosimilar pathway was established. In this pathway, “biosimilarity” to an approved reference product must be demonstrated through an extensive comparability exercise. This exercise includes physicochemical studies, appropriate nonclinical studies, limited pharmacokinetic and pharmacodynamics studies, and comparative clinical studies to establish efficacy and safety (European Medicines agency, London 2006). The United States (US) Food and Drug Administration (FDA) has proposed a stepwise approach for providing totality of evidence of similarity between a proposed biosimilar product and a US-licensed (reference) product. This stepwise approach starts with the assessment of critical quality attributes that are relevant to clinical outcomes in structural and functional characterization in manufacturing process of the proposed biosimilar product. The FDA suggests that these critical quality attributes be identified first and then classified into three tiers depending upon their criticality: most (Tier 1), mild to moderate (Tier 2), and least (Tier 3) relevant to clinical outcomes [143]. However, even after demonstrating comparability, the products might not be similar in terms of risk of immunogenicity. Therefore, a detailed immunogenicity assessment is still warranted.

## 6. Conclusion 

Recent years have seen an expansion in the development and manufacturing of protein therapeutic drugs, both in terms of number of molecules and in terms of global production capacity. In this review, we discussed the causes of immunogenicity which could be product-related (inherent property of the product or might be picked up during the manufacturing process), patient-related, or linked to the route of administration. We also discussed the impact of PTMs of therapeutic proteins on immunogenicity; and it is clear that some PTMs lead to increased immunogenicity. Managing immunogenicity in both preclinical and clinical settings is very important. With the advent of novel analytical technologies, there has been a dramatic enhancement of the capability to analyze and characterize therapeutics. Also, analysis of these proteins* in vivo* is critical to understand biological effects of PTMs. Relevant human immune system-specific animal models are now being established to study these biological effects. Future studies should focus on the development of sensitive diagnostics that can predict immunogenicity-mediated adverse events in small fraction of subjects that develop clinically relevant ADAs and hence mitigate the risk due to unwarranted immunogenicity.

## Figures and Tables

**Table 1:** Immunogenicity of FDA-approved biologics.

SI number	Biologic	Type/target	Indications	Immunogenicity (% patients)		Reference
				Binding antibodies	Neutralizing antibodies	
(1)	Adalimumab	Human IgG1 antibody specific for TNF alpha	Rheumatoid arthritis, psoriasis	5%–89%	5%–89%	[24–27]

(2)	Alemtuzumab	Humanized monoclonal antibody which binds to CD52 on leukocytes	B cell chronic lymphocytic leukaemia	30%–70%	30%–70%	[28]

(3)	Belimumab	Human monoclonal antibody that inhibits the biologic activity of the soluble form of the essential B cell survival factor B-lymphocyte stimulator	Systemic lupus erythematosus	4.8%	3/11	[29] European Medicines Agency Assessment Report (http://www.ema.europa.eu/docs/en_GB/document_library/EPAR_-_Public_assessment_report/human/002015/WC500110152.pdf)

(4)	Canakinumab	Human monoclonal antibody anti-IL-1*β*	Inflammatory diseases related to cryopyrin-associated periodic syndromes (familial cold autoinflammatory syndrome and Muckle-Wells syndrome)	0%	0%	[24, 30]

(5)	Cetuximab	Human/mouse chimeric monoclonal antibody that binds specifically to the extracellular domain of the human epidermal growth factor receptor	Colorectal cancer, squamous cell carcinoma of the head and neck	5%	Data not available	Drug label (http://pi.lilly.com/us/erbitux-uspi.pdf)

(6)	Denosumab	Human monoclonal antibody RANK ligand inhibitor	Treatment of postmenopausal women with osteoporosis at high risk for fracture To increase bone mass in men at high risk for fracture receiving androgen deprivation therapy for nonmetastatic prostate cancer To increase bone mass in women at high risk for fracture receiving adjuvant aromatase inhibitor therapy for breast cancer	<1%	0%	Drug label (http://www.accessdata.fda.gov/drugsatfda_docs/label/2011/125320s5s6lbl.pdf)

(7)	Golimumab	Human monoclonal antibody anti-TNF-alpha	Rheumatoid arthritis, psoriatic arthritis, and ankylosing spondylitis	4%	Data not available	[31–33]

(8)	Infliximab	Chimeric monoclonal antibody TNF blocker	Crohn's disease, ulcerative colitis, rheumatoid arthritis, ankylosing spondylitis, and psoriasis	Crohn's Disease: 10%, psoriatic arthritis: 15% psoriasis: 36–51%	Data not available	Drug label (http://www.accessdata.fda.gov/drugsatfda_docs/label/2013/103772s5359lbl.pdf)

(9)	Ipilimumab	Human monoclonal antibody that binds to cytotoxic T-lymphocyte-associated antigen 4 (CTLA-4)	Melanoma	1.1% of 1024 evaluable patients tested positive for binding antibodies and 6.9% of 58 evaluable patients, who were treated with 0.3 mg/kg (dose cohort with the lowest trough levels) dose, tested positive for binding antibodies.	0%	Drug label (http://www.accessdata.fda.gov/drugsatfda_docs/label/2013/103772s5359lbl.pdf)

(10)	Natalizumab	Human monoclonal antibody, which works against the cell adhesion molecule *α*4-integrin	Multiple sclerosis and Crohn's disease	9%	9%	Drug label (http://www.accessdata.fda.gov/drugsatfda_docs/label/2008/125104s033lbl.pdf)

(11)	Nivolumab	Human monoclonal antibody IgG4 PD-1 immune checkpoint inhibitor	Squamous non-small cell lung cancer, unresectable or metastatic melanoma, and disease progression following ipilimumab and, if BRAF V600 mutation positive, a BRAF inhibitor	8.5%	0.7%	[34] Drug label (http://www.accessdata.fda.gov/drugsatfda_docs/label/2014/125554lbl.pdf)

(12)	Ofatumumab	Human monoclonal antibody anti-CD20	Rheumatoid arthritis, chronic lymphocytic leukaemia	0%	0%	[24, 35]

(13)	Panitumumab	Human monoclonal antibody against the epidermal growth factor receptor	Colorectal carcinoma	1–4.6%	0.8–1.6%	[24, 36–38] Drug label (http://www.accessdata.fda.gov/drugsatfda_docs/label/2009/125147s080lbl.pdf)

(14)	Pembrolizumab	Human monoclonal antibody PD-1 blocking	Unresectable or metastatic melanoma and disease progression following ipilimumab and, if BRAF V600 mutation positive, a BRAF inhibitor, metastatic NSCLC whose tumors express PD-L1	0.3%	0.3%	[39] Drug label (http://www.accessdata.fda.gov/drugsatfda_docs/label/2015/125514s004s006lbl.pdf)

(15)	Ramucirumab	Human monoclonal antibody vascular endothelial growth factor receptor 2 antagonist	Gastric cancer	6%	1%	Drug label (http://www.accessdata.fda.gov/drugsatfda_docs/label/2014/125477s002lbl.pdf)

(16)	Rituximab	Chimeric monoclonal antibody that targets the CD20 molecule expressed on the surface of B cells	Non-Hodgkin's lymphoma, rheumatoid arthritis	11%	Data not available	[40]

(17)	Secukinumab	Human monoclonal antibody against IL-17A	Plaque psoriasis	0.4%	3/10	[41] Drug information (http://www.fda.gov/downloads/AdvisoryCommittees/CommitteesMeetingMaterials/Drugs/DermatologicandOphthalmicDrugsAdvisoryCommittee/UCM419023.pdf)

(18)	Siltuximab	Chimeric monoclonal antibody anti-IL-6	Multicentric Castleman's disease (a rare lymphoproliferative disorder) being human immunodeficiency virus-negative and human herpes virus-8-negative	0.2%	0%	Drug label (http://www.accessdata.fda.gov/drugsatfda_docs/label/2014/125496s000lbl.pdf)

(19)	Ustekinumab	Human monoclonal antibody that binds to the p40 protein subunit used by both the IL-12 and IL-23 cytokines.	Psoriasis	6.6%	Data not available	[42]

(20)	Vedolizumab	Humanised IgG1 monoclonal antibody that binds to the human *α*4*β*7 integrin	Ulcerative colitis and Crohn's disease	4%	33/56	Drug label (http://www.accessdata.fda.gov/drugsatfda_docs/label/2014/125476s000lbl.pdf)

IgG: immunoglobulin G; IL: interleukin; TNF: tumour necrosis factor; CD: cluster of differentiation; RANK: receptor activator of nuclear factor kappa-B; PD-1: programmed cell death protein-1; PDL-1: programmed death ligand-1; NSCLC: non-small cell lung cancer.
